# Tautomerism in 11-Hydroxyaklavinone: A DFT Study

**DOI:** 10.1100/2012/526289

**Published:** 2012-05-01

**Authors:** Lemi Türker

**Affiliations:** Department of Chemistry, Middle East Technical University, 06531 Ankara, Turkey

## Abstract

The antharquinone-based chromophore of 11-hydroxyaklavinone is present in the structure of an anticancer agent, daunomycin. On the other hand, aklavinone is the parent aglycone of certain anthracycline antibiotics that possess anti-cancer activity too. The structures of aklavinone and its 11-hydroxy derivative have many –OH groups, and two keto groups which may take place in certain tautomeric equilibria. Of these tautomeric forms, presently the one involving the anthraquinone based tautomers of 11-hydroxyaklavinone has been investigated quantum chemically in the framework of the density functional theory at the levels of RB3LYP/6-31G(d) and RB3LYP/6-31G(d,p).

## 1. Introduction

 Aklavinone [1] exists as the parent aglycone of two families of glycosidically derived anthracycline antibiotics that possess significant anticancer activity [1, 2]. The backbone of the chromophore system of 11-hydroxyaklavinone is common with the chromophore moiety of daunomycin, an anti-cancer agent. Note that one of the phenolic –OH groups of 11-hydroxyaklavinone is in the methyl ether form in daunomycin. Also it is worth mentioning that alicyclic rings of daunomycin and 11-hydroxyaklavinone possess different substituents.

The isolation, characterization, and biological activity of seven antibiotics which contain aklavinone as the aglycone were published by Oki et al. [3, 4]. Of these antibiotics, aclacinomycin A was the subject of substantial investigation and was found to have high anticancer activity while being less toxic than the clinically useful rhodomycin, daunorubicin [3–7]. Because of its biological importance, there was high interest in its total synthesis [8–11]. Jones and Lock managed the synthesis of aklavinone via electron-deficient o-quinoid pyrones [12]. Certain aklavinone derivatives were obtained with the reduction of daunomycin and 7-deoxydaunomycin [13]. An electron spin resonance study on quinone-containing carcinostatics, aclacinomycin A, and its derivatives was reported [14]. The hyperfine structure of ESR spectra was satisfactorily reproduced by simulations, using the hyperfine coupling constants obtained by the INDO molecular orbital method [14].

Isotopic labeling experiments have shown that daunomycin is synthesized in *Streptormyces galilaeus* from a tetra cyclic precursor, 11-hydroxyaklavinone, and aklavinone in turn is synthesized from acetate [15].

Tautomerism in 7-deoxyalklavinone has been mentioned in the literature [16]. Daunomycin, which contains aklavinone type-kernel (basically 11-hydroxyaklavinone backbone), is known to have antitumor action. It has been shown that the mechanism of the biological effect of daunomycin is due to its ability to set between pairs of DNA bases [17]. Its chromophore (11-hydroxyaklavinone-like moiety) sets DNA pairs apart to find its place in between them [17].

 11-Hydroxyaklavinone having various –OH groups and two keto groups of quinoid type may exhibit many tautomeric forms, which may shed some light on the tautomeric equilibria that might occur in daunomycin. Presently, some tautomers of 11-hydroxyaklavinone have been investigated quantum chemically.

## 2. Method

 The initial geometry optimizations of all the structures leading to energy minima were achieved by using the MM2 (molecular mechanics) method followed by the semiempirical PM3 self-consistent fields molecular orbital (SCF MO) method [18, 19] at the restricted level [20]. Then, the geometry optimizations were achieved by using various restricted Hartree-Fock (RHF) methods successively and finally optimizing within the framework of the density functional theory (DFT, RB3LYP) [21, 22] at the level of 6–31G(d) (pseudo potential option, LACVP*) and RB3LYP/6-31G(d,p). It is known that RB3LYP/6-31G(d)-and RB3LYP/6-31G(d,p)-type calculations produce good results for many purposes including the ground state and transition state geometries and thermochemistry [23, 24]. Note that the exchange term of B3LYP consists of hybrid Hartree-Fock and local spin density (LSD) exchange functions with Becke's gradient correlation to LSD exchange [25]. The correlation term of B3LYP consists of the Vosko-Wilk-Nusair (VWN3) local correlation functional [26] and Lee-Yang-Parr (LYP) correlation correction functional [27]. Also B3LYP/6-31G(d)-(pseudo potential option] type calculations have been performed presently to enlighten the transition state geometries and energies for a certain pair of the tautomers.

For each set of calculations, vibrational analyses were done (using the same basis set employed in the corresponding geometry optimizations). The normal mode analysis for each structure yielded no imaginary frequencies for the *3N-6* vibrational degrees of freedom, where *N* is the number of atoms in the system. This indicates that the structure of each molecule corresponds to at least a local minimum on the potential energy surface. For the transition state geometry calculations the presence of single imaginary frequency has been confirmed. All these computations were performed by using the Spartan 06 [28] package program.

## 3. Results and Discussion

**Figure 1:**
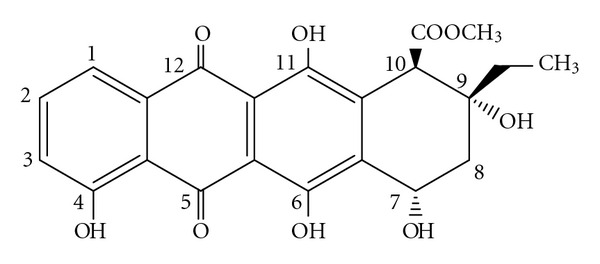
Numbering of carbons in 11-hydroxyaklavinone.

**Figure 2:**
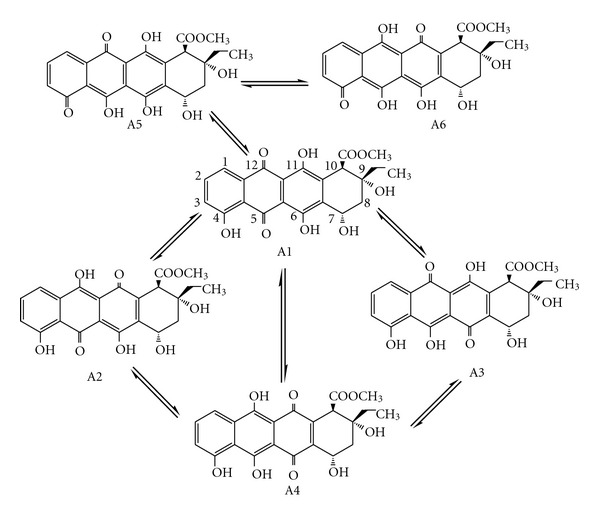
Tautomers presently considered.

 The structure and numbering of 11-hydroxyaklavinone are shown in Figure 1. As seen there, it contains phenolic as well as alcoholic –OH groups in addition to keto groups. Chromophoric part can be considered as trihydroxy anthraquinone moiety. The positions of the phenolic –OH groups enable one to write down various tautomeric forms, at the expense of aromatic nature of the phenolic rings. However, in some forms of the tautomers, one or two aromatic sextets are retained.  Figure 2 shows the various tautomers of 11-hydroxyaklavinone (A1), which are interconvertible via 1,3 and 1,5-type tautomeric routs. In the tautomers A2, A3, and A5, a single phenolic ring happens whereas in A1 and A4 the skeletons contain 8,9- and 1,4-anthraquinone moiety having three phenolic –OH groups, respectively. In A6, all these –OH groups are enolic, which have been originally phenolic in A1 structure. In 11-hydroxyaklavinone (A1) the two aromatic sextets are isolated Clar's sextes, whereas in A4 an embedded naphthalene like structure allows Clar's sextet to shift from one phenolic ring to the other. Therefore, the stabilities of these tautomers, especially the stability of A4, are to be worth considering theoretically. The tautomers considered seem to be capable of forming intramolecular hydrogen bonding, which may affect the stabilities, thus complicating the picture.

**Figure 3:**
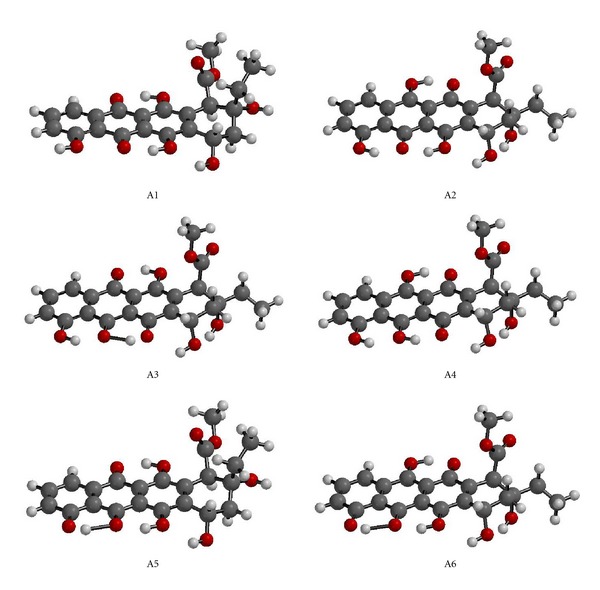
The geometry optimized structures of the tautomers (RB3LYP/6–31G(d)).

Figure 3 shows the geometry optimized structures of the 11-hydroxyaklavinone and its tautomers presently considered. As seen in the figure, in A3, A5, and A6 hydrogen bonding is clearly seen between the –OH group and the keto group next to it. On the alicyclic ring, the orientation of the ester methoxy group is different in A2–A4 and A6 from A1 and A5. Also the conformation of the ethyl group varies from one tautomer to the other.

Table 1 shows the total electronic energies of the species considered. The stability order is A3 > A6 > A2 > A4 > A5 > A1 (RB3LYP/6-31G(d)) in vacuum conditions. The calculations indicate that this order is somewhat changed in aqueous solution, that is, A6 > A2 > A4 > A3 > A5 > A1 (see Table 2). In each series A1 appears to be the least stable structure even though it contains two aromatic sextets in the structure. Although A6 does not have any aromatic ring, the electronic effects arising from better extended conjugation and hydrogen bonding capability compared to A1 could be the cause of A6 to have more negative total energy in aqueous medium. A4 has a Clar's sextet similar to naphthalene and possesses medium stability among the tautomers. 

When one compares the stability orders in the vacuum and aqueous conditions the effect of solvation/hydrogen bonding appears to be not negligible. Many examples exist in the literature supporting the importance of solvents on the tautomeric equilibria. An example related to the 11-hydroxyaklavinone and aklavinone is the 9-anthrone/9-anthranol equilibrium where the equilibrium lies practically on the side of the keto form in the gas phase and inert solvents but the enol form is favored in protic solvents [29]. Also local dipole-dipole interactions may contribute to the whole stability of each tautomer. RB3LYP/6–31G(d,p) type calculations produce the stability order (in vacuum) as A3 ~ A2 ~ A6 > A4 ~ A5 > A1 and in aqueous conditions as A6 ~ A2 > A4 > A1 > A3 > A5. Both the 6-31G(d)-and 6–31G(d,p)-based DFT calculations indicate that A3 is the most stable tautomer in the vacuum and A1 is the least stable one (RB3LYP/6-31+G(d)-type calculations carried on A1, A3, and A6 indicate that the stabilities of these three tautomers in the vacuum follow the order of A3 > A6 > A1 and in water A6 > A3 > A1), whereas in aqueous conditions A6 becomes the most stable (RB3LYP/6–31G(d)-and RB3LYP/6–31G(d,p)) tautomer and the least stable ones are A1 and A6 in the cases of 6–31G(d) and 6–31G(d,p)-based calculations, respectively.

On the other hand, the transition state energies calculated at the presently employed level of calculations are shown in Table 3 and the order of these energies indicates that A1 → A3 transition state is the most, whereas A2 → A4 is the least, energetic transition.

The standard free energy of formation values calculated at the level of B3LYP/6–31G(d) (pseudo potential) are shown in Table 4. Irrespective of the basis sets used, the order from the lowest (the most negative G° value) to the highest is A3 < A6 < *A*2 < A4 < A5 < A1, which is the order of total energies in the vacuum conditions. The order is indicative of the fact that A3 is the most favorable and A1 is the least likely in terms of free energy of formation criterion.

The free energy of the tautomeric change between any two tautomeric forms is tabulated in Table 5. According to the results of calculations, the most favorable change is A1 → A3 and then A1 → A2, whereas the least likely one among the tautomers considered is A3 → A4.

**Figure 4:**
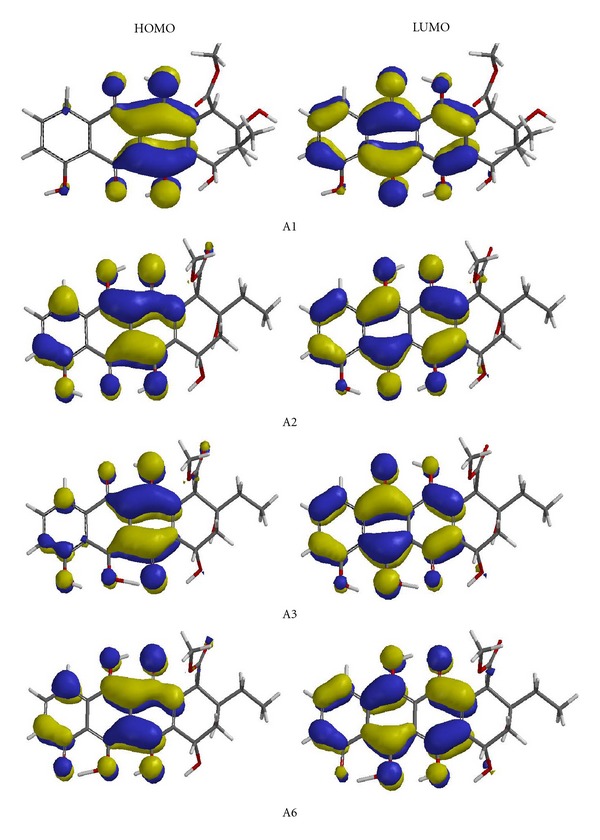
The HOMO and LUMO patterns of some of the tautomers (RB3LYP/6–31G(d)).

Table 6 shows the HOMO, LUMO, and the interfrontier molecular orbital energy gap (Δ*ε*) values of the species of concern. Tautomers A3 and A4 are characterized with the lowest and highest lying HOMO energy values, respectively. Irrespective of the basis sets used, the order is A3 < A5 < A6 < A2 < A1 < A4, whereas the order of LUMO energies is A6 < A2 < A4 < A3 < A5 < A1, which is coincidentally the same with the order of stabilities (6–31G(d)) in aqueous solution. As for the Δ*ε* values, A1 has the largest energy gap whereas A4 possesses the smallest one with either of the basis sets used. The energies given in Table 6 is of course in the realm of the density functional theory (DFT). Note that early work in the field tended to resist any attempt to interpret Kohn-Sham orbitals of DFT. However, later on it has been observed that Kohn-Sham orbitals are compatible with Hartree-Fock (HF) orbitals [30]. The shapes of Kohn-Sham orbitals tend to be remarkably similar to canonical HF molecular orbitals. Moreover, HF virtual orbitals tend to be too high in energy and anomalously diffuse compared to Kohn-Sham virtual orbitals. However, approximate functionals are quite bad at predicting ionization potentials in this fashion without applying some sort of correction scheme, for example, an empirical linear scaling of the eigenvalues [30].  Figure 4 shows the HOMO and LUMO patterns of some of the tautomers. Note that A3 and A6 are the most stable tautomers in the vacuum and in aqueous solution, respectively, whereas A1 is the least stable in both cases of the basis sets used.

As seen in Figure 4, the HOMO and LUMO of the tautomers exhibit *π*-symmetry and, mainly constructed by the contribution of atomic orbitals of the part originating from anthraquinone moiety of A1.

**Figure 5:**
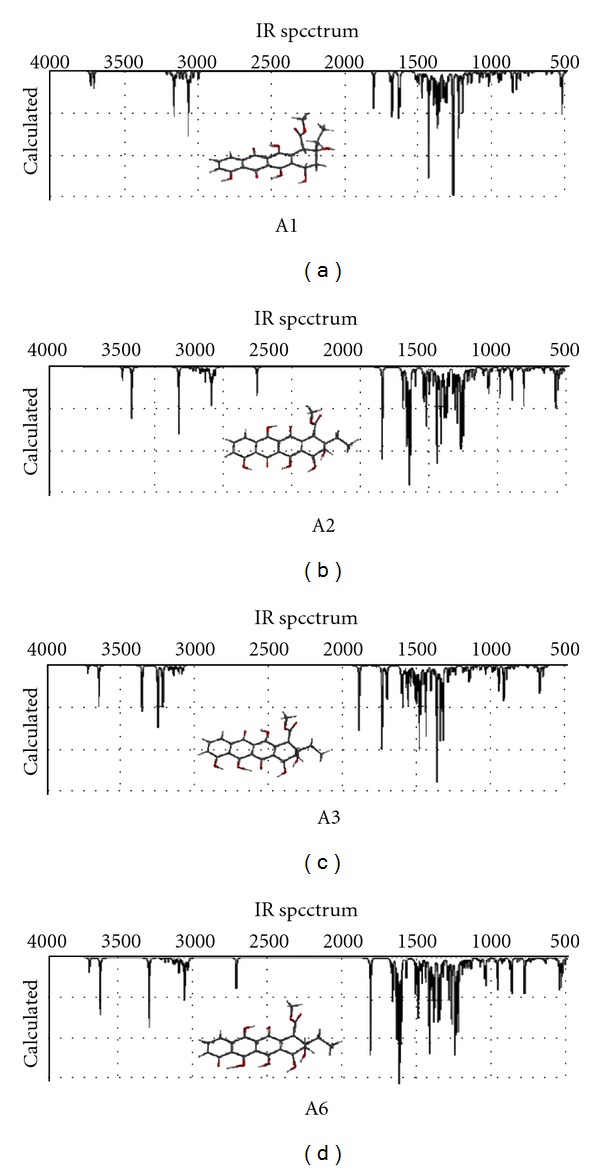
IR spectra of some of the tautomers (RB3LYP/6–31G(d)).

Figure 5 shows the calculated IR spectra of some selected tautomers. The alcoholic and phenolic –OH stretchings occur above 3500 cm^−1^, whereas the saturated and unsaturated C = O stretchings happen in the region of 1800–1600 cm^−1^ having varying strengths.

## 4. Conclusion

 Of the various tautomeric forms of 11-hydroxyaklavinone presently considered, A3 is the most favorable in terms of the free energy of formation from its elements. It is also the most stable one in the vacuum conditions whereas in aqueous medium A6 far precedes A3 in the stability. 11-Hydroxyaklavinone (A1) is either the least stable or has medium stability in the order depending on the basis set used. However, the energies of the tautomers are not very different from each other. According to the Gibbs free energy change, A1 should turn into A3 which is also kinetically favored conversion within the constraints of the computational approach employed presently.

## Figures and Tables

**Table 1:** Total energy (au) values of the structures considered.

Structure	RB3LYP/6–31G(d)		RB3LYP/6–31G(d,p)	
	Total energy	Corrected total energy	Total energy	Corrected total energy
A1	−1527.42	−1527.02	−1527.47	−1527.07
A2	−1527.44	−1527.04	−1527.49	−1527.10
A3	−1527.45	−1527.05	−1527.50	−1527.10
A4	−1527.44	−1527.04	−1527.49	−1527.09
A5	−1527.44	−1527.04	−1527.49	−1527.09
A6	−1527.44	−1527.04	−1527.49	−1527.10

**Table 2:** Total energy (au) values of the structures considered in aqueous medium.

Structure	RB3LYP/6–31G(d)		RB3LYP/6–31G(d,p)	
	Total energy	Corrected total energy	Total energy	Corrected total energy
A1	−1527.45	−1527.06	−1527.56	−1527.16
A2	−1527.53	−1527.14	−1527.59	−1527.20
A3	−1527.47	−1527.07	−1527.52	−1527.13
A4	−1527.53	−1527.13	−1527.58	−1527.18
A5	−1527.46	−1527.07	−1527.52	−1527.12
A6	−1527.54	−1527.14	−1527.59	−1527.20

**Table 3:** Transition state energies.

Transition state	Total energy (au)	Corrected total energy (au)
A1-A2	−1527.4161	−1527.0204
A1–A3	−1527.4108	−1527.0154
A1–A5	−1527.4334	−1527.0373
A2–A4	−1527.4352	−1527.0430
A5-A6	−1527.4364	−1527.0401

**Table 4:** G° and relative G° (kJ/mol) values of the structures considered.

Structure	RB3LYP/6–31G(d)		RB3LYP/6–31G(d,p)	
	G°	Rel G°	G°	Rel G°
A1	−4009341.81	82.77	−4009474.40	84.60
A2	−4009405.41	19.17	−4009539.59	19.41
A3	−4009424.58	0	−4009559.00	0
A4	−4009395.43	29.15	−4009529.11	29.89
A5	−4009392.24	32.34	−4009524.75	34.25
A6	−4009407.42	17.16	−4009541.43	17.57

**Table 5:** G° and ΔG_ij_° values (kJ/mol) of the structures considered (RB3LYP/6–31G(d)).

Ai → Aj	G_i_°	G_j_°	ΔG_ij_°
A1→A2	−4009341.81	−4009405.41	−63.6
A1→A3	−4009341.81	−4009424.58	−82.77
A1→A4	−4009341.81	−4009395.43	−53.62
A1→A5	−4009341.81	−4009392.24	−50.43
A2→A4	−4009405.41	−4009395.43	9.98
A3→A4	−4009424.58	−4009395.43	29.15
A5→A6	−4009392.24	−4009407.42	−15.18

**Table 6:** The HOMO and LUMO energies (eV) and interfrontier energy gaps (Δ*ε*) of the structures considered.

Structure	RB3LYP/6–31G(d)			RB3LYP/6–31G(d,p)		
	HOMO	LUMO	Δ*ε*	HOMO	LUMO	Δ*ε*
A1	−5.93	−2.99	2.93	−5.91	−3.00	2.91
A2	−5.99	−3.44	2.55	−5.99	−3.44	2.55
A3	−6.22	−3.31	2.90	−6.21	−3.32	2.90
A4	−5.92	−3.37	2.55	−5.92	−3.38	2.54
A5	−6.15	−3.24	2.91	−6.14	−3.25	2.89
A6	−6.00	−3.44	2.55	−6.00	−3.44	2.55
